# Supplementary material for: Health care workers in conflict and post-conflict settings: Systematic mapping of the evidence
Source: PLoS One. 2020 May 29;15(5):e0233757. doi: 10.1371/journal.pone.0233757 (PMC7259645; doi:10.1371/journal.pone.0233757)
Supplement: S1 File — (DOCX) [file pone.0233757.s001.docx]

**Supplementary file 1: Electronic databases search strategies**

**Medline search strategy**

Database: Ovid MEDLINE(R) Epub Ahead of Print, In-Process & Other Non-Indexed Citations, Ovid MEDLINE(R) Daily and Ovid MEDLINE(R) <1946 to Present>

Search Strategy:

--------------------------------------------------------------------------------

1 exp health personnel/ or exp laboratory personnel/ or exp health manpower/ (461359)

2 ((health or healthcare or medical or hospital? or nursing or social) adj2 (worker? or professional? or personnel or manpower or staff or workforce)).tw. (182451)

3 (doctor? or physician? or nurse or nurses or midwife or midwives or midwifery or mid-wife or mid-wifery or mid-wives or paramedic? or medic or medics or pharmacist?).tw. (666533)

4 ((lab or laboratory) adj technician?).tw. (1322)

5 students, medical/ or students, nursing/ (48357)

6 (medical adj (resident or residents or graduate? or student?)).tw. (36356)

7 (nursing adj (student? or graduate?)).tw. (12407)

8 (birth adj attendant?).tw. (2016)

9 (human adj resource?).tw. (7950)

10 or/1-9 (1107597)

11 exp Warfare/ (35699)

12 ((conflict* or combat) adj2 (area? or zone? or setting? or region or regions or military or armed or ethnic or country or countries or state or states or field? or recovery)).tw. (3830)

13 (armed-conflict or armed-conflicts or battlefield or battlefields or battle-field or battle-fields or warfare or war or wars or wartime or warzone or incursion or insurrection).tw. (43035)

14 (post-conflict? or postconflict? or post-war? or postwar? or (post adj (war or wars or conflict?))).tw. (3331)

15 (health adj (system? or service?) adj (reconstruction or reconstructing or rehabilitation or rehabilitating or rebuilding)).tw. (26)

16 (peace-building or peace-keeping or peacekeeping or peace-making).tw. (420)

17 or/11-16 (68291)

18 10 and 17 (8862)

19 limit 18 to yr="1990 -Current" (7094)

20 limit 18 to yr="2000 -Current" (5327)

***************************

**Embase search strategy**

Database: Embase <1980 to 2017 Week 27>

Search Strategy:

--------------------------------------------------------------------------------

1 exp health care manpower/ or exp health care personnel/ or exp laboratory personnel/ (1225938)

2 ((health or healthcare or medical or hospital? or nursing or social) adj2 (worker? or professional? or personnel or manpower or staff or workforce)).tw. (221284)

3 (doctor? or physician? or nurse or nurses or midwife or midwives or midwifery or mid-wife or mid-wifery or mid-wives or paramedic? or medic or medics or pharmacist?).tw. (822984)

4 ((lab or laboratory) adj technician?).tw. (1657)

5 students, medical/ or students, nursing/ (61850)

6 (medical adj (resident or residents or graduate? or student?)).tw. (42195)

7 (nursing adj (student? or graduate?)).tw. (11612)

8 (birth adj attendant?).tw. (1841)

9 (human adj resource?).tw. (9071)

10 or/1-9 (1742974)

11 exp war/ (28732)

12 ((conflict* or combat) adj2 (area? or zone? or setting? or region or regions or military or armed or ethnic or country or countries or state or states or field? or recovery)).tw. (4113)

13 (armed-conflict or armed-conflicts or battlefield or battlefields or battle-field or battle-fields or warfare or war or wars or wartime or warzone or incursion or insurrection).tw. (42091)

14 (post-conflict? or postconflict? or post-war? or postwar? or (post adj (war or wars or conflict?))).tw. (3073)

15 (health adj (system? or service?) adj (reconstruction or reconstructing or rehabilitation or rehabilitating or rebuilding)).tw. (19)

16 (peace-building or peace-keeping or peacekeeping or peace-making).tw. (448)

17 or/11-16 (58831)

18 10 and 17 (10044)

19 limit 18 to yr="1990 -Current" (8789)

20 limit 18 to yr="2000 -Current" (7046)

***************************

**CENTRAL search strategy**

'((health manpower) or (health personnel) or (laboratory personnel) or (health worker*) or (health professional*) or (health staff) or (health workforce) or (healthcare personnel) or (healthcare manpower) or (healthcare worker*) or (healthcare professional*) or (healthcare staff) or (healthcare workforce) or (medical personnel) or (medical manpower) or (medical worker*) or (medical professional*) or (medical staff) or (medical workforce) or (hospital personnel) or (hospital manpower) or (hospital worker*) or (hospital professional*) or (hospital staff) or (hospital workforce) or (hospitals personnel) OR (hospitals manpower) or (hospitals worker*) or (hospitals professional*) or (hospitals staff) or (hospitals workforce) or (social worker*) or doctor or doctors or physician* or nurse or nurses or midwife or midwives or midwifery or mid-wife or mid-wifery or mid-wives or paramedic* or medic or medics or pharmacist* or (lab technician) or (lab technicians) or (laboratory technician) or (laboratory technicians) or (medical resident) or (medical residents) or (medical graduate) or (medical graduates) or (medical student) or (medical students) or (nursing students) or (nursing student) or (nursing graduate) or (nursing graduates) or (birth attendant) or (birth attendants) or (human resource) or (human resources)) in Title, Abstract, Keywords and (warfare or war or wars or warzone or wartime or conflict* or armed-conflict* or combat or battlefield* or battle-field or battle-fields or incursion or insurrection or post-conflict* or postconflict* or post-war or post-wars or postwar or postwars) in Title, Abstract, Keywords , Publication Year from 1990 to 2017

**CINAHL search strategy**

S17 S9 AND S16

S16 S10 OR S11 OR S12 OR S13 OR S14 OR S15

S15 TI ((health N1 (service* or system*)) N1 (reconstruction or reconstructing or rehabilitation or rehabilitating or rebuilding)) OR AB ((health N1 (service* or system*)) N1 (reconstruction or reconstructing or rehabilitation or rehabilitating or rebuilding)) OR KW ((health N1 (service* or system*)) N1 (reconstruction or reconstructing or rehabilitation or rehabilitating or rebuilding))

S14 TI (peace-building or peace-keeping or peacekeeping or peace-making) OR AB (peace-building or peace-keeping or peacekeeping or peace-making) OR KW (peace-building or peace-keeping or peacekeeping or peace-making)

S13 TI (post N1 conflict*) OR AB (post N1 conflict*) OR KW (post N1 conflict*)

S12 TI (post-conflict* or postconflict* or post-war* or postwar*) OR AB (post-conflict* or postconflict* or post-war* or postwar*) OR KW (post-conflict* or postconflict* or post-war* or postwar*)

S11 TI ((conflict* or combat) N2 (area or areas or zone or zones or setting* or region or regions or military or armed or ethnic or country or countries or state or states or field or fields or recovery)) OR AB ((conflict* or combat) N2 (area or areas or zone or zones or setting* or region or regions or military or armed or ethnic or country or countries or state or states or field or fields or recovery)) OR KW ((conflict* or combat) N2 (area or areas or zone or zones or setting* or region or regions or military or armed or ethnic or country or countries or state or states or field or fields or recovery))

S10 TI (armed-conflict or armed-conflicts or battlefield or battlefields or battle-field or battle-fields or warfare or war or wars or wartime or warzone or incursion or insurrection) OR AB (armed-conflict or armed-conflicts or battlefield or battlefields or battle-field or battle-fields or warfare or war or wars or wartime or warzone or incursion or insurrection) OR KW (armed-conflict or armed-conflicts or battlefield or battlefields or battle-field or battle-fields or warfare or war or wars or wartime or warzone or incursion or insurrection)

S9 S1 OR S2 OR S3 OR S4 OR S5 OR S6 OR S7 OR S8

S8 TI (human N1 resource*) OR AB (human N1 resource*) OR KW TI (human N1 resource*) Search modes - Boolean/Phrase

S7 TI (birth N1 attendant*) OR AB (birth N1 attendant*) OR KW (birth N1 attendant*)

S6 TI (nursing N1 (student* or graduate*)) OR AB (nursing N1 (student* or graduate*)) OR KW (nursing N1 (student* or graduate*))

S5 TI (medical N1 (resident or residents or graduate* or student*)) OR AB (medical N1 (resident or residents or graduate* or student*)) OR KW (medical N1 (resident or residents or graduate* or student*))

S4 TI ((lab or laboratory) N1 technician*) OR AB ((lab or laboratory) N1 technician*) OR KW ((lab or laboratory) N1 technician*)

S3 (doctor or doctors or physician* or nurse or nurses or midwife or midwives or midwifery or mid-wife or mid-wifery or mid-wives or paramedic or paramedics or medic or medics or pharmacist*) OR AB (doctor or doctors or physician* or nurse or nurses or midwife or midwives or midwifery or mid-wife or mid-wifery or mid-wives or paramedic or paramedics or medic or medics or pharmacist*) OR KW (doctor or doctors or physician* or nurse or nurses or midwife or midwives or midwifery or mid-wife or mid-wifery or mid-wives or paramedic or paramedics or medic or medics or pharmacist*)

S2 TI ((health or healthcare or medical or hospital* or nursing or social) N2 (worker* or professional* or personnel or manpower or staff or workforce)) Or AB ((health or healthcare or medical or hospital* or nursing or social) N2 (worker* or professional* or personnel or manpower or staff or workforce)) OR KW ((health or healthcare or medical or hospital* or nursing or social) N2 (worker* or professional* or personnel or manpower or staff or workforce))

S1 TI (human N1 resource?) OR AB (human N1 resource?) OR KW TI (human N1 resource?)

**HRH Global Resource Center search strategy**

**In the website** [**https://www.hrhresourcecenter.org/**](https://www.hrhresourcecenter.org/)**, we browsed by subject heading to** [**Fragile Environments**](http://www.hrhresourcecenter.org/taxonomy_menu/1/38)

Fragile environments refer to “health workers and HRH in unstable areas due to political unrest, war, natural disasters, etc.”

- [Conflict Affected Regions](http://www.hrhresourcecenter.org/taxonomy_menu/1/38/39)
  health workers and HRH areas affected by political unrest and war
- [Natural Disaster Affected Regions](http://www.hrhresourcecenter.org/taxonomy_menu/1/38/207)
  health workers and HRH areas affected by natural disasters such as earthquakes, hurricanes, etc.

**ReBUILD Consortium Search Strategy**

**In the website** <https://rebuildconsortium.com/> we chose “Resources” then we filtered by Human Resources for Health (under Research Projects) and by Health Workers (under Themes).
