# Supplementary material for: Health care workers in conflict and post-conflict settings: Systematic mapping of the evidence
Source: PLoS One. 2020 May 29;15(5):e0233757. doi: 10.1371/journal.pone.0233757 (PMC7259645; doi:10.1371/journal.pone.0233757)
Supplement: S2 File — (DOCX) [file pone.0233757.s002.docx]

**Supplementary file 2: Definitions of themes**

Themes related to conflict setting:

- Violence against health care workers: refers to direct attacks and violent acts against health care workers such as killing, arrest and kidnapping.
- Education: refers to training and education (e.g. continuing medical education) received by professionals in conflict zones.
- Practicing in conflict setting: refers to the special practices (e.g. clinical) and conditions under which health workers practice in conflict setting.
- Migration: refers to the issue of migration, movement and exodus of health care workers from conflict zones.
- Other

Themes of the study for the post-conflict settings:

- Workforce supply: refers to recruitment and selection, pay and pre-service education and training.
- Workforce distribution: refers to deployment and incentives.
- Workforce performance: refers to work organization and job design, management and supervision, performance appraisal, performance-related incentives, in-service training.
- Other
