# Supplementary material for: Health care workers in conflict and post-conflict settings: Systematic mapping of the evidence
Source: PLoS One. 2020 May 29;15(5):e0233757. doi: 10.1371/journal.pone.0233757 (PMC7259645; doi:10.1371/journal.pone.0233757)
Supplement: S3 File — (PDF) [file pone.0233757.s003.pdf]

Health care workers in conflict and post-conflict settings: systematic mapping of the evidence

Geographical map of the included papers in conflict (N=325) and post-conflict (N=170) settings

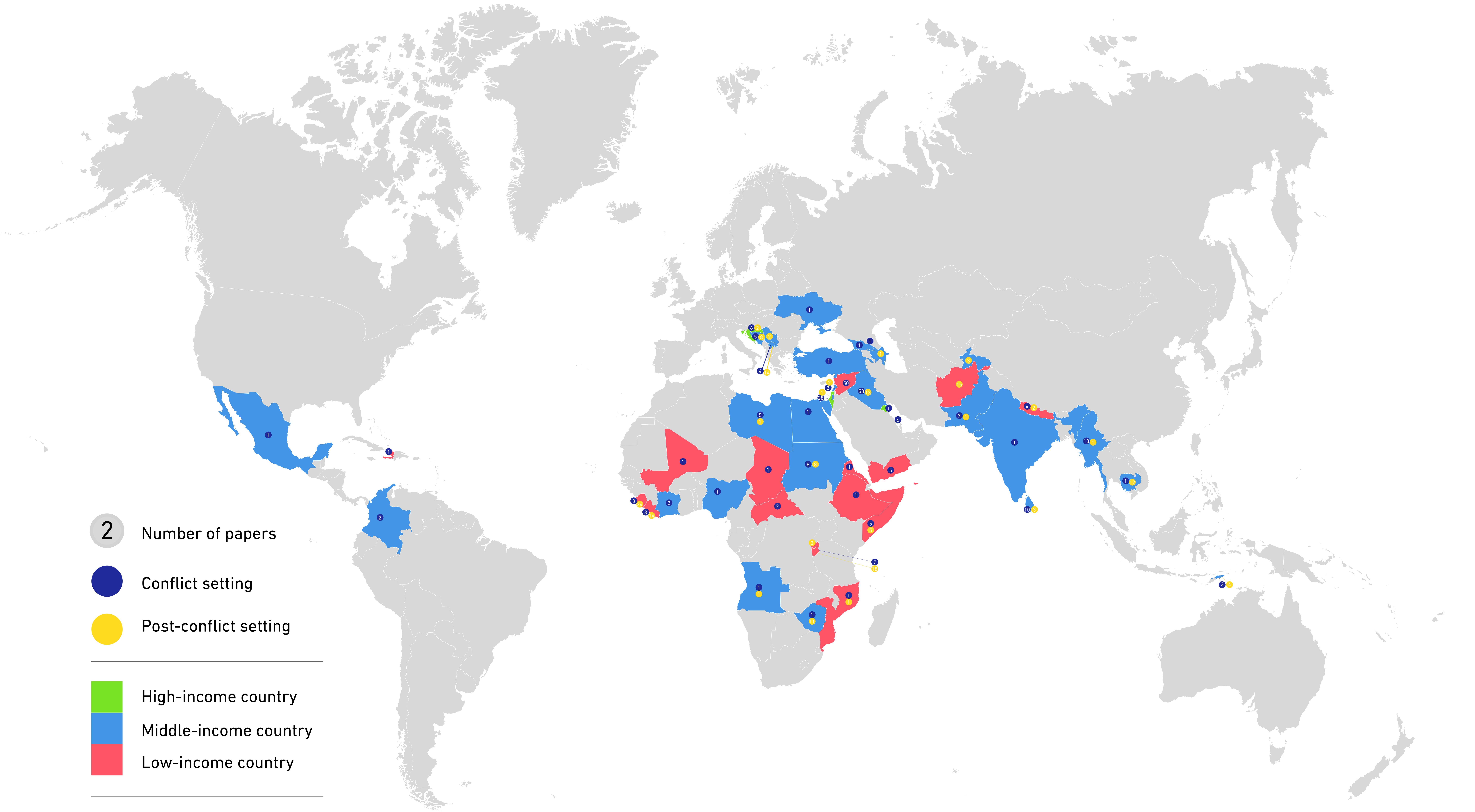

One paper may address more than one country.

67 papers were not about a specific conflict setting and 16 papers were not about a specific post-conflict setting
