# Supplementary material for: Health care workers in conflict and post-conflict settings: Systematic mapping of the evidence
Source: PLoS One. 2020 May 29;15(5):e0233757. doi: 10.1371/journal.pone.0233757 (PMC7259645; doi:10.1371/journal.pone.0233757)
Supplement: Attachment — Submitted filename: PONE-D-19-29429_reviewer SSQ.pdf [file pone.0233757.s005.pdf]

PLOS ONE

Health Care Workers in Conflict and Post-Conflict Settings: Systematic Mapping of the Evidence

--Manuscript Draft--

**Manuscript Number:**	PONE-D-19-29429
**Article Type:**	Research Article
**Full Title:**	Health Care Workers in Conflict and Post-Conflict Settings: Systematic Mapping of the Evidence
**Short Title:**	Health Care Workers in Conflict and Post-Conflict Settings
**Corresponding Author:**	Elie A. Akl<br>American University of Beirut<br>Beirut, LEBANON
**Keywords:**	Health care workers; Health workforce; Health personnel; Armed conflict; Warfare; Post-conflict; Systematic mapping
**Abstract:**	Background: Health care workers (HCWs) are essential for the delivery of health care services in conflict areas and in rebuilding health systems post-conflict.<br>Objective: The aim of this study was to systematically identify and map the published evidence on HCWs in conflict and post-conflict settings.<br>Methods: We conducted a systematic mapping of the literature. We included a wide range of study designs, addressing any type of personnel providing health services in either conflict or post-conflict settings. We conducted a descriptive analysis of the general characteristics of the included papers and built two interactive systematic maps organized by country, study design and theme.<br>Results: Out of 13,863 identified citations, we included a total of 474 studies: 304 on conflict settings, 149 on post-conflict settings, and 21 on both conflict and post-conflict settings. For conflict settings, the most studied counties were Iraq (15%), Syria (15%), Israel (10%), and Palestine (9%). The most common types of publication was opinion pieces in conflict settings (39%), and primary studies (33%) in post-conflict settings. In addition, most of the first and corresponding authors were affiliated with countries different from the country subject of the paper. The majority of papers in both conflict and post conflict settings did not report funding sources (81% and 53%), conflicts of interest of authors (73% and 62%) and around half of primary studies did not report on ethical approvals (45% and 41%).<br>Conclusions: This systematic mapping provide a comprehensive database of evidence about HCWs in conflict and post-conflict settings on a global scale. It can inform policymakers, funders and researchers working in the field of health care workers in conflict and post-conflict settings.
**Order of Authors:**	Lama Bou-Karroum
	Amena El-Harakeh
	Inas Kassamany
	Hussein Ismail
	Nour El Arnaout
	Rana Charide
	Farah Madi
	Sarah Jamali
	Tim Martineau
	Fadi El-Jardali
	Elie A. Akl
**Additional Information:**	
**Question**	**Response**

Powered by Editorial Manager® and ProduXion Manager® from Aries Systems Corporation

**Financial Disclosure**<br><br>Enter a financial disclosure statement that describes the sources of funding for the work included in this submission. Review the submission guidelines for detailed requirements. View published research articles from *PLOS ONE* for specific examples.<br><br>This statement is required for submission and **will appear in the published article** if the submission is accepted. Please make sure it is accurate.<br><br>**Unfunded studies**<br>Enter: *The author(s) received no specific funding for this work.*<br><br>**Funded studies**<br>Enter a statement with the following details:<br>• Initials of the authors who received each award<br>• Grant numbers awarded to each author<br>• The full name of each funder<br>• URL of each funder website<br>• Did the sponsors or funders play any role in the study design, data collection and analysis, decision to publish, or preparation of the manuscript?<br>• **NO** - Include this sentence at the end of your statement: *The funders had no role in study design, data collection and analysis, decision to publish, or preparation of the manuscript.*<br>• **YES** - Specify the role(s) played.<br><br>* typeset	EAA and FEJ received funding from the Lebanese National Council for Scientific Research (CNRS) and the Alliance for Health Policy and Systems Research of the World Health Organization (WHO). The funders had no role in study design, data collection and analysis, decision to publish, or preparation of the manuscript.
**Competing Interests**<br><br>Use the instructions below to enter a competing interest statement for this submission. On behalf of all authors, disclose any competing interests that could be perceived to bias this work—acknowledging all financial support and any other relevant financial or non-financial competing interests.<br><br>This statement **will appear in the published article** if the submission is accepted. Please make sure it is	The authors have declared that no competing interests exist.

accurate. View published research articles from *PLOS ONE* for specific examples.<br><br>**NO authors have competing interests**<br><br>Enter: *The authors have declared that no competing interests exist.*<br><br>**Authors with competing interests**<br><br>Enter competing interest details beginning with this statement:<br><br>*I have read the journal's policy and the authors of this manuscript have the following competing interests: [insert competing interests here]*<br><br>* typeset	
**Ethics Statement**<br><br>Enter an ethics statement for this submission. This statement is required if the study involved:<br><br>• Human participants<br>• Human specimens or tissue<br>• Vertebrate animals or cephalopods<br>• Vertebrate embryos or tissues<br>• Field research<br><br>Write "N/A" if the submission does not require an ethics statement.<br><br>General guidance is provided below. Consult the submission guidelines for detailed instructions. **Make sure that all information entered here is included in the Methods section of the manuscript.**	N/A

**Format for specific study types**<br><br>**Human Subject Research (involving human participants and/or tissue)**<br>• Give the name of the institutional review board or ethics committee that approved the study<br>• Include the approval number and/or a statement indicating approval of this research<br>• Indicate the form of consent obtained (written/oral) or the reason that consent was not obtained (e.g. the data were analyzed anonymously)<br><br>**Animal Research (involving vertebrate animals, embryos or tissues)**<br>• Provide the name of the Institutional Animal Care and Use Committee (IACUC) or other relevant ethics board that reviewed the study protocol, and indicate whether they approved this research or granted a formal waiver of ethical approval<br>• Include an approval number if one was obtained<br>• If the study involved *non-human primates*, add *additional details* about animal welfare and steps taken to ameliorate suffering<br>• If anesthesia, euthanasia, or any kind of animal sacrifice is part of the study, include briefly which substances and/or methods were applied<br><br>**Field Research**<br><br>Include the following details if this study involves the collection of plant, animal, or other materials from a natural setting:<br>• Field permit number<br>• Name of the institution or relevant body that granted permission	
**Data Availability**<br><br>Authors are required to make all data underlying the findings described fully available, without restriction, and from the time of publication. PLOS allows rare exceptions to address legal and ethical concerns. See the PLOS Data Policy and FAQ for detailed information.	Yes - all data are fully available without restriction

Powered by Editorial Manager® and ProduXion Manager® from Aries Systems Corporation

A Data Availability Statement describing where the data can be found is required at submission. Your answers to this question constitute the Data Availability Statement and **will be published in the article**, if accepted.<br><br>**Important:** Stating 'data available on request from the author' is not sufficient. If your data are only available upon request, select 'No' for the first question and explain your exceptional situation in the text box.<br><br>Do the authors confirm that all data underlying the findings described in their manuscript are fully available without restriction?	
**Describe where the data may be found in full sentences. If you are copying our sample text, replace any instances of XXX with the appropriate details.**<br><br>• If the data are **held or will be held in a public repository**, include URLs, accession numbers or DOIs. If this information will only be available after acceptance, indicate this by ticking the box below. For example: *All XXX files are available from the XXX database (accession number(s) XXX, XXX.).*<br>• If the data are all contained **within the manuscript and/or Supporting Information files**, enter the following: *All relevant data are within the manuscript and its Supporting Information files.*<br>• If neither of these applies but you are able to provide **details of access elsewhere**, with or without limitations, please do so. For example:<br><br>*Data cannot be shared publicly because of [XXX]. Data are available from the XXX Institutional Data Access / Ethics Committee (contact via XXX) for researchers who meet the criteria for access to confidential data.*<br><br>*The data underlying the results presented in the study are available from (include the name of the third party*	All relevant data are within the manuscript and its Supporting Information files.

and contact information or URL).<br>• This text is appropriate if the data are owned by a third party and authors do not have permission to share the data.<br><br>* typeset	
Additional data availability information:	

Manuscript

Click here to access/download;Manuscript;HCWs in conflict and post-conflict settings_20191022.docx

Health care workers in conflict and post-conflict settings: systematic mapping of the evidence

Lama Bou-Karroum[1,2], Amena El-Harakeh[1,3], Inas Kassamany[2], Hussein Ismail[4], Nour El Arnaout[5], Rana Charide[4], Farah Madi[4], Sarah Jamali[6], Tim Martineau[7], Fadi El-Jardali[1,2,8], Elie A. Akl[1,3,7,8]*

Affiliations

[1] Center for Systematic Reviews on Health Policy and Systems Research (SPARK), American University of Beirut, Beirut, Lebanon

[2] Department of Health Management and Policy, Faculty of Health Sciences, American University of Beirut, Beirut, Lebanon

[3] Clinical Research Institute (CRI), American University of Beirut Medical Center, Beirut, Lebanon

[4] Faculty of Health Sciences, American University of Beirut, Beirut, Lebanon

[5] Global Health Institute, American University of Beirut, Beirut, Lebanon

[6] Faculty of Medicine, American University of Beirut, Beirut, Lebanon

[7] Department of International Public Health, Liverpool School of Tropical Medicine, United Kingdom

[8] Department of Health Research Methods, Evidence, and Impact (HEI), McMaster University, Hamilton, Ontario, Canada

[8] Department of Internal Medicine, Faculty of Medicine, American University of Beirut, Beirut, Lebanon

*Corresponding author

E-mail: ea32@aub.edu.lb (EAA)

Abstract

Background: Health care workers (HCWs) are essential for the delivery of health care services in conflict areas and in rebuilding health systems post-conflict.

Objective: The aim of this study was to systematically identify and map the published evidence on HCWs in conflict and post-conflict settings.

Methods: We conducted a systematic mapping of the literature. We included a wide range of study designs, addressing any type of personnel providing health services in either conflict or post-conflict settings. We conducted a descriptive analysis of the general characteristics of the included papers and built two interactive systematic maps organized by country, study design and theme.

Results: Out of 13,863 identified citations, we included a total of 474 studies: 304 on conflict settings, 149 on post-conflict settings, and 21 on both conflict and post-conflict settings. For conflict settings, the most studied counties were Iraq (15%), Syria (15%), Israel (10%), and Palestine (9%). The most common types of publication was opinion pieces in conflict settings (39%), and primary studies (33%) in post-conflict settings. In addition, most of the first and corresponding authors were affiliated with countries different from the country subject of the paper. The majority of papers in both conflict and post conflict settings did not report funding sources (81% and 53%), conflicts of interest of authors (73% and 62%) and around half of primary studies did not report on ethical approvals (45% and 41%).

Conclusions: This systematic mapping provide a comprehensive database of evidence about HCWs in conflict and post-conflict settings on a global scale. It can inform policymakers, funders and researchers working in the field of health care workers in conflict and post-conflict settings.

Introduction

Health care workers (HCWs) are essential for the delivery of health care services in conflict areas and in rebuilding health systems post-conflict. However, HCWs in conflict areas around the world are being threatened, detained, and killed. For instance, in Syria, Physicians for Humans Rights has reported that, since the start of the conflict till December 2017, 847 medical personnel have been killed [1]. In Afghanistan, around 92 attacks against health facilities and health workers killed 14 health workers and four caretakers in the period extending from March 1, 2015 till February 10, 2016 [2].

Direct attacks and the insecurity have led to the exodus of HCWs from conflict areas. In Syria, 50% of the health workers and 95% of physicians living in Aleppo have left the country since 2011. In Iraq, almost half of the health professionals have emigrated since 2014 [2]. In Nigeria, almost all health workers have escaped areas controlled by Boko Haram since 2012, leading to the closure of 450 health facilities [2].

The resulting shortage of HCWs has devastating effects on the delivery of, and access to health care not only during conflicts but also after. The post-conflict settings are characterized by poor health outcomes due to limited availability of HCWs and the disruption of health systems [3, 4]. Rebuilding the health care workforce is critical to address health needs and strengthen health systems. Furthermore, post-conflict settings present a window of opportunity to develop responsive and evidence-informed strategies and policies to address defects in the supply, distribution and performance of the health workforce [4, 5].

In 2012, the World Health Organization (WHO) passed a resolution that calls on the WHO Director General for leadership in documenting evidence of attacks against health workers, facilities, and patients in

situations of armed conflict [6]. A scoping review on HCWs in Syria and other "Arab Spring" countries showed scarcity of research evidence on HCWs in the setting of the "Arab Spring" [7]. While that review revealed a number of themes of interest (e.g., violence against health care workers, education, practicing in conflict setting, migration), it focused on only one region and did not address post-conflict settings. Therefore, the objective of this study was to systematically identify and map the published evidence on HCWs in both conflict and post-conflict settings.

Methods

Study design

This systematic mapping was based on a protocol registered with Open Science Framework [8]. We followed standard methodology for the screening, data extraction and coding, and data analysis and visualizing the findings in systematic mapping. Contrary to systematic reviews, systematic mapping does not aim to answer a specific question but instead "collates, describes and catalogues available evidence (e.g. primary, secondary, quantitative or qualitative) relating to a topic of interest" [9]. The studies included in a systematic map can be used to identify evidence for policy-relevant questions, knowledge gaps to direct future primary research, and knowledge clusters. Knowledge clusters are sub-sets of evidence that may be suitable for secondary research, for example systematic review.

Eligibility criteria

Population of interest: Our population of interest consisted of any personnel providing health services such as: midwives, nurses, paramedics, pharmacists, physicians, laboratory technicians,

community health workers as well as medical students and trainees. We excluded military HCWs because we aimed to focus on the delivery of health care primarily to civilians.

Setting of interest: We ~~also~~ included both conflict and post-conflict settings. We considered both conflicts between and within states [10]. We focused on contemporary conflicts that started after or were ongoing in the 1990s. Post-conflict settings are considered as a stage of recovery of the state from conflict or crisis and a stage of rebuilding and reconstruction starting from emergency and stabilization followed by transition and recovery, and peace and development [5, 11, 12].

Study design: We included all types of study designs, including news, editorials/ commentaries/ opinion pieces, technical reports, primary studies, narrative reviews, and systematic reviews. We excluded conference abstracts. We restricted our eligibility criteria to papers published after the year 2000 to better reflect the current challenges facing health systems and the new aspects of contemporary conflicts.

Literature search

We searched the following electronic databases: Medline (Ovid), PubMed, EMBASE (Ovid), the Cochrane Central Register of Controlled Trials (CENTRAL), Cumulative Index of Nursing and Allied Health Literature CINAHL (EBSCOT) on July 2017. We also searched the ReBUILD Consortium Resources webpage and the Human Resources for Health (HRH) Global Resource Center.

We used both index terms and free text words for the two following concepts: (1) health care workers and (2) conflict and post-conflict settings. The search terms and Medical Subject Headings (MeSH) terms for each database were developed with the guidance of an information specialist.

We did not limit the search to specific languages. S1 Appendix provides the search strategies for the different databases.

Selection process

Title and abstract screening: Teams of two reviewers used the above eligibility criteria to screen titles and abstracts of identified citations in duplicate and independently for potential eligibility. We retrieved the full text for citations judged as potentially eligible by at least one of the two reviewers.

Full-text screening: Teams of two reviewers used the above eligibility criteria to screen the full texts in duplicate and independently for eligibility. The teams of two reviewers resolved disagreement by discussion or with the help of a third reviewer. We used standardized and pilot tested screening forms. We conducted calibration exercises to ensure the validity of the selection process.

Data extraction and coding

Two reviewers extracted data using standardized and pilot tested forms. The reviewers resolved any disagreement by discussion and when needed with the help of a third reviewer. We conducted calibration exercises to ensure the validity of the data abstraction process.

We extracted from each paper the following information:

- Citation;
- Year of publication;
- Countr(ies) subject of the paper;

- Type of publication (e.g., news, editorial, correspondence, opinion pieces, primary study, narrative review, systematic review, case study, technical report)
- Language of publication;
- Authors' information:
  - Total number of authors;
  - Number of authors from the countr(ies) subject of the paper;
  - Country of affiliation of the first author;
  - Country of affiliation of contact author;
- Characteristics of the journal of publication (name and impact factor);
- Setting (conflict or post-conflict);
- Theme(s) of the study for the conflict settings: we adopted the themes from a previous scoping review on health care workers in the setting of Arab Spring [7]: violence against health care workers, education, practicing in conflict setting, migration, and other (S2 Appendix);
- Theme(s) of the study for the post-conflict settings: we adopted the theme(s) from a previous review on human resource management in post-conflict health systems [5]: workforce supply, workforce distribution, workforce performance; and other (S2 Appendix);
- Reporting of funding of the study;
- Reporting of conflict of interest of authors;
- Ethical approval of the study.

Critical appraisal

We did not appraise the quality of included studies since our review is consistent with standard systematic mapping methodology [9].

Data analysis

We conducted a descriptive analysis of the general characteristics of the included papers using frequencies. We also used the results of this review to build two interactive and visual systematic evidence maps on (1) HCWs in conflict settings and (2) HCWs in post-conflict settings. We represented the evidence maps by country, type of publication and themes. We have also provided direct links to the included studies.

Results

Study selection

Fig 1 summarizes the study selection process (S1 Fig). Out of 13,863 identified unique citations, we included a total of 474 studies: 304 on conflict settings, 149 on post-conflict settings, and 21 on both conflict and post-conflict settings. We excluded 968 papers for the following reasons: not study design of interest (n=63); not setting of interest (n=324); not population of interest (n=538); and not timeframe of interest (e.g. ceasefire was called on before 1990) (n=43). We present below our findings on the characteristics of the included papers, journals, authors, funding, conflicts of interest, and ethics reporting. We also report on the two generated systematic maps.

Fig 1. Preferred reporting items for systematic reviews and meta-analyses (PRISMA) study flow diagram for selection.

Characteristics of the included papers

Table 1 presents the countries subject of the included papers. For conflict settings (N=325), 79% of the included papers addressed specific conflicts related to 47 countries. The most studied counties were Iraq (15%), Syria (15%), Israel (10%), and Palestine (9%). For post-conflict settings (N=170), 91% of the included papers addressed specific settings related to 32 countries. Sierra Leone (14%) was the most studied country followed by Uganda (11%) and Afghanistan (9%). The majority of papers on conflict and post-conflict were published in English language (98% and 100% respectively).

Table 1 Countries subject of the included papers in conflict (N=325) and post-conflict (N=170) settings

**Conflict (N=325)**		**Post-conflict (N=170)**	
Countries subject of papers*	n (%)	Countries subject of papers*	n (%)
Not specific	67 (21)	Sierra Leone	24 (14)
Iraq	50 (15)	Uganda	19 (11)
Syria	50 (15)	Afghanistan	16 (9)
Israel	32 (10)	Not specific	16 (9)
Palestine	28 (9)	Kosovo	14 (8)
Afghanistan	16 (5)	Liberia	14 (8)
Myanmar	13 (4)	Iraq	13 (7)
Sri Lanka	10 (3)	Cambodia	11 (6)
Congo	9 (3)	Zimbabwe	11 (6)
Sudan	8 (2)	Bosnia and Herzegovina	8 (5)
Pakistan	7 (2)	Sudan	8 (5)
Bahrain	6 (2)	Congo	4 (2)
Croatia	6 (2)	Timor-Leste	4 (2)
Uganda	6 (2)	Israel	3 (2)
Bosnia and Herzegovina	5 (2)	Sri Lanka	3 (2)
Libya	5 (2)	Somalia	3 (2)
Somalia	5 (2)	Other countries**	20 (12)
Yemen	5 (2)		
Other countries**	47 (14)		

*One paper may address more than one country.
** Countries that were reported in 1% of the papers or less were grouped under “other countries”.

Table 2 represents the themes focus of the papers on health care workers in conflict (N=325) and post-conflict (N=170) settings. More than one theme was reported in 33% of the papers on HCWs in conflict settings and in 52% of the papers on HCWs in post-conflict settings. In addition to the themes about HCWs in conflict settings reported in Bou-Karroum et al. (2018) [7], three additional themes emerged in this review, and those were the role of HCWs in peace promotion or protecting health care, mental health of HCWs, and medical ethics. Most of the included papers on conflict setting addressed the theme of violence against health workers (41%), followed by health or medical practice (34%) and education (21%). For post-conflict settings, besides the themes adopted from Roome et al. (2014) [5], an emerging theme was the mental health of HCWs. The majority of the included papers on post-conflict setting addressed the workforce performance theme (77%) followed by workforce supply (58%).

Table 2 Topics focus of the papers on health care workers in conflict (N=325) and post-conflict (N=170) settings

**Topics of the papers***	n (%)
**Conflict setting (N=325)**	
• Violence against health care workers	133 (41)
• Health or medical practice	109 (34)
• Education	67 (21)
• Role in peace promotion or protecting health care	48 (15)
• Mental health	42 (13)
• Migration	37 (11)
• Medical ethics	16 (5)
**Post-conflict setting (N=170)**	
• Workforce performance	131 (77)
• Workforce supply	98 (58)
• Workforce distribution	40 (24)
• Retention	22 (13)
• Mental health	8 (5)

*One paper may address more than one topic.

Fig 2 shows the annual production rate of the included papers (S2 Fig). The year for the peak number of publications was 2013 for conflict settings and 2014 for post-conflict settings. Fig 3 shows the types of publication of the included papers (S3 Fig). Opinion pieces represented the most common type of publication (39%) in conflict settings, followed by primary studies (23%) and news (18%). Primary studies were the most common type of publication (33%) in post-conflict settings followed by technical reports (24%) and case studies (21%).

Fig 2. Publication year of articles of the papers included in the systematic maps in conflict (N=325) and post-conflict settings*

Fig 3. Types of publication* of the included papers on health care workers in in conflict (N=325) and post-conflict (N=170) settings

Characteristics of the journals

For conflict settings (N=325), the included papers were published across 134 journals. The journals that published the highest proportions of included studies were the Lancet (15%), BMJ (7%), and CMAJ (5%). Out of the 134 journals, 90 journals (67%) had 2017 impact factors. 230 of the 325 papers were published in these 90 journals and had a median impact factor of 4.74 (IQR = 1.74-27.94).

For post-conflict settings (N=170), the included papers were published across 79 journals. The journals that published the highest number of included studies were the Lancet (6%), Conflict and Health (5%), and Health Policy and Planning (5%). Out of the 79 journals, 51 journals (65%) had 2017 impact factors. 92 of the 170 papers were published in these 51 journals and had a median impact factor of 2.42 (IQR = 1.61- 3.31).

Characteristics of the authors

Table 3 summarizes the characteristics of the authors of the included papers. For conflict settings, 69% of the included papers reported affiliations of authors and addressed specific conflict(s). Out of these, 40% had at least 1 author affiliated with the country subject of the paper. The median percentage of authors affiliated with country subject of the paper was null (0%) (IQR =0-75). In addition, most of the first and corresponding authors were affiliated with countries different from the country subject of the paper (68% and 70% respectively), mainly the United States of America (42% and 39% respectively) followed by the United Kingdom (21% and 24% respectively).

Table 3 Characteristics of authors of the included papers in conflict (N=325) and post-conflict (N=170) settings

**Any/all authors**	**Conflict (N=325)**	**Post-conflict (N=170)**
	**n (%)**	
Papers with named authors	302 (93)	161 (95)
Papers reporting affiliations of authors	279 (86)	141 (83)
	**N=224* (69)**	**N=128* (75)**
Papers with at least 1 author affiliated with country subject of paper	90 (40)	68 (53)
% authors affiliated with country subject of paper (median [IQR])	0 (0-75)	20 (0-67)
**First author**	**N=224**	**N=128**
Country subject of paper	68 (30)	42 (33)
Different country	151 (68)	84 (66)
- United States of America	63 (42)	27 (32)
- United Kingdom	31 (21)	31 (37)
- European countries other than UK	20 (13)	7 (8)
- Canada	15 (10)	7 (8)
- Other	22 (14)	12 (15)
Independent	5 (2)	2 (1)
**Corresponding author**	**N=213§**	**N=125§§**
Country subject of paper	60 (28)	29 (23)
Different country	148 (70)	94 (75)
- United States of America	58 (39)	29 (31)
- United Kingdom	35 (24)	38 (40)
- European countries other than UK	20 (14)	8 (9)

- Canada	14 (9)	7 (7)
- Other	21 (14)	12 (13)
Independent	5 (2)	2 (2)

Abbreviation: IQR, interquartile range
* This is the number of papers reporting affiliations of authors and addressing a specific setting.
§ The corresponding author was unclear in 11 papers.
§§ The corresponding author was unclear in 3 papers.

For post-conflict settings, 75% of the included papers reported affiliations of authors and addressed specific conflict(s). Out of these, about half (53%) had at least 1 author affiliated with the country subject of the paper. The median percentage of authors affiliated with the country subject of the paper was 20% (IQR = 0-67). Similar to conflict settings, most of the first and corresponding authors were affiliated with countries different from the country subject of the paper (66% and 75% respectively), mainly the United Kingdom (37% and 40% respectively) followed by the United States of America (32 and 31% respectively).

Funding, conflicts of interest and ethics reporting characteristics

Table 4 shows the funding, conflicts of interest, and ethics reporting characteristics of the included papers. For conflict settings, most of the included papers did not report funding sources (81%) or statements of conflicts of interest of authors (73%). Out of the included primary studies, about half (55%) reported ethical approval to conduct the studies. For post-conflict settings, about half of the included papers did not report funding sources (53%) and 62% did not report on the conflicts of interest of authors. Out of the included primary studies, 59% reported ethical approval.

Table 4 Funding, conflicts of interest and ethics reporting characteristics of the included papers in conflict (N=325) and post-conflict (N=170) settings

	Conflict (N=325)	Post-conflict (N=170)
**Funding sources**	n (%)	
Not reported	263 (81)	90 (53)
Reported as funded	50 (15)	75 (44)
Reported as not funded	12 (4)	5 (3)
**Conflicts of interest**		
Not reported	238 (73)	106 (62)
Reported	87 (27)	64 (38)
**Ethical approval of primary studies**	**Conflict (N=76)**	**Post-conflict (N=56)**
Not reported	33 (44)	21 (38)
Reported as approved	42 (55)	33 (59)
Reported as not required	1 (1)	2 (3)

Systematic maps

The two systematic maps, which represent a visual and interactive overview of the evidence on health care workers in conflict and post-conflict settings, can be freely accessed and downloaded using the following links for conflict settings (http://evidencemaphcw.com/gapmap/conflict) and for post-conflict settings (http://evidencemaphcw.com/gapmap/post-conflict). The maps allow data to be filtered and sorted by type of primary studies (experimental, survey, qualitative, mixed-methods, and document analysis). The maps contain links that redirect the user to PubMed or other databases, to access the title and abstract of included papers, when available.

The systematic map for conflict settings shows that papers on violence and attacks against HCWs were mainly not country specific, about Syria, or about Iraq. The theme of health or medical practice of HCWs was mainly addressed in Iraq and Syria. Education and training of health care workers was the theme mainly addressed in Iraq and Myanmar. Primary studies on HCWs in

conflict setting were mainly about Israel and Palestine with a focus on mental health in both countries.

The systematic map for post-conflict settings shows that the theme of workforce performance was mainly about Sierra Leone and Uganda. The papers on workforce supply were mainly not country specific, about Afghanistan, or about Sierra Leone. Primary studies on HCWs in post-conflict setting were mainly about Sierra Leone and Afghanistan with a focus on workforce performance in both countries.

Discussion

This review presents a systematic mapping of the evidence on health care workers in conflict and post-conflict settings. It has uncovered interesting findings relating to the characteristics of the included papers, journals, and authors respectively; as well as the reporting of funding, conflicts of interest, and ethics.

The systematic map in conflict settings shows the scarcity of primary studies conducted in conflict settings with the predominance of news, opinion pieces and commentaries. This is in line with a previous scoping review on health care workers in the setting of Arab Spring that showed the scarcity of research evidence [7]. Similarly, Patel et al. reported on the lack of baseline and routine data mainly on violence against health workers [13]. In contrast to conflict settings, primary studies represented the most frequent type of publications on health care workers in post-conflict settings. These findings might relate to the specific settings in which the conflict and post conflict studies were conducted. However, they might also reflect the challenges to conducting primary research

in conflict settings, including security concerns, difficulties in obtaining representative samples and with data collection, political bias, lack of tools and methods specific to conflict settings, and insufficient research funding and capacity [1, 13, 14].

The majority of authors (including first and corresponding) of the included papers were affiliated with high income counties, as opposed to being affiliated with the country subject of the paper. This finding may reflect global imbalances in research capacity between high and low and middle income countries [15-17]. Reasons for this imbalance include limited funding, instability, poor research training, collaboration challenges, and shortage of skilled human resources in low and middle income countries [1, 16-20].

The majority of included papers on conflict setting addressed the theme of violence against health workers. They mainly related to the conflicts in Iraq and Syria. Similarly, the previously published scoping review focusing on health care workers in the setting of Arab Spring found that violence was the most tackled themes [7]. In the post-conflict setting, the most addressed themes were workforce performance followed by workforce supply. These findings concur with a previous review by Roome et al. on human resources management in post-conflict health systems [5]. This shows the need for more studies on the topic of workforce distribution, which is important to ensure equity in health service provision.

Another interesting finding is the low rates of reporting of funding sources and disclosures of conflict of interest by the authors of the included studies. This is particularly for papers about HCWs in conflict settings. Indeed, funders may have specific agendas while researchers may have

political biases and tendency to take sides [14]. These may lead to distorted research and biased data that could be used to mislead local and international communities and negatively affecting policy making. Reporting of funding sources and conflict of interest becomes important to better assess the confidence in the publication, particularly when it reports primary studies or makes policy recommendations.

We also found a relatively low reporting of ethical approvals for primary studies, in both conflict and post-conflict settings. This might be attributed to a weak local research capacity including the absence of or complicated ethical review boards [1]. Reporting and seeking ethical approvals in these settings is important given the vulnerability of individuals living in conflict-affected states [21]. This calls journals publishing research conducted in conflict settings to have stringent policies for reporting funding, conflict of interest and ethical approval.

To our knowledge, this is the first systematic mapping of evidence on health care workers in conflict and post-conflict settings. One strength of this study is that we have followed a standardized methodology for conducting and reporting systematic mapping [9]. Further, we have used published frameworks to classify studies on HCWs in conflict and post-conflict settings [5, 7]. One limitation of this study is restricting inclusion to studies published after the year 2000. However, studies published before 2000 might not reflect the current challenges facing health systems and the new aspects of contemporary conflicts.

The findings of this review and the resulting systematic maps can support policy makers working on rebuilding health systems post-conflict. These systematic maps provide a comprehensive

resource of evidence about HCWs in conflict and post-conflict settings on a global scale. As such, policymakers as well as researchers can use them to find relevant studies by theme. In addition, the mapped evidence can inform policies and practices to protect, support and address the needs of the workers in conflict settings. The evidence identified can also inform efforts and strategies for reconstruction and rebuilding of post-conflict health systems in particular human resource for health.

The findings also highlight the need to strengthen the capacity of local researchers working in conflict-affected states. Also, they can inform the agendas of funders and researchers working in the field of health care workers in conflict and post-conflict settings of potential knowledge gaps. This systematic map will inform areas for potential systematic reviews in the field, and maybe provide a jumpstart for those reviews, given the relevant studies have been already identified and organized by theme.

Acknowledgement

We would like to thank Ms. Nour Hemadi for helping in the screening process and Ms. Karen Bou-Karroum, Ms. Rand Al Ghoussaini and Mr. Mark Jreij for helping in the data abstraction process.

References

1. Woodward A, Sheahan K, Martineau T, Sondorp E. Health systems research in fragile and conflict affected states: a qualitative study of associated challenges. Health Research Policy and Systems. 2017;15(1):44. doi: 10.1186/s12961-017-0204-x.

2. Safeguarding Health in Conflict. No Protection No Respect: Health Workers and Health Facilities Under Attack 2015 and Early 2016. Published on 23 May 2016. https://reliefweb.int/report/world/no-protection-no-respect-health-workers-and-health-facilities-under-attack-2015-and

3. Rubenstein RJHLS. Health in Postconflict and Fragile States. United States Institute of Peace. 2012;Special Report 301.

4. Martineau T, McPake B, Theobald S, Raven J, Ensor T, Fustukian S, et al. Leaving no one behind: lessons on rebuilding health systems in conflict- and crisis-affected states. BMJ Global Health. 2017;2(2):e000327. doi: 10.1136/bmjgh-2017-000327.

5. Roome E, Raven J, Martineau T. Human resource management in post-conflict health systems: review of research and knowledge gaps. Conflict and health. 2014;8:18. Epub 2014/10/09. doi: 10.1186/1752-1505-8-18. PubMed PMID: 25295071; PubMed Central PMCID: PMCPMC4187016.

6. World Health Assembly (WHA). WHO's response, and role as the health cluster lead, in meeting the growing demands of health in humanitarian emergencies. 2012. http://apps.who.int/gb/ebwha/pdf_files/WHA65/A65_R20-en.pdf

7. Bou-Karroum L, Daou KN, Nomier M, El Arnaout N, Fouad FM, El-Jardali F, et al. Health Care Workers in the setting of the "Arab Spring": a scoping review for the Lancet-AUB

Commission on Syria. J Glob Health. 2019;9(1):010402-. doi: 10.7189/jogh.09.010402. PubMed PMID: 30410745.

8. Khamis AM, Hakoum MB, Bou-Karroum L, Habib JR, Ali A, Guyatt G, et al. Requirements of health policy and services journals for authors to disclose financial and non-financial conflicts of interest: a cross-sectional study. Health research policy and systems. 2017;15(1):80. doi: 10.1186/s12961-017-0244-2.

9. James KL, Randall NP, Haddaway NR. A methodology for systematic mapping in environmental sciences. Environmental Evidence. 2016;5(1):7. doi: 10.1186/s13750-016-0059-6.

10. International Committee of the Red Cross (ICRC). How is the term "Armed Conflict" defined in international humanitarian law? 2008. https://www.icrc.org/en/doc/resources/documents/article/other/armed-conflict-article-170308.htm

11. McPake B. Health systems in conflict affected states - are they different from in other low and middle income countries? Early ideas from the work of the ReBUILD programme. ReBuild Consortium. 2017.

12. Ahonsi BA. Towards more informed responses to gender violence and HIV/AIDS in post-conflict West African settings: Nordiska Afrikainstitutet; 2010.

13. Patel P, Gibson-Fall F, Sullivan R, Irwin R. Documenting attacks on health workers and facilities in armed conflicts. Bull World Health Organ. 2017;95(1):79-81. Epub 08/30. doi: 10.2471/BLT.15.168328. PubMed PMID: 28053368.

14. Sen K, Hussain H, Al-Faisal W. Ethics in times of conflict: some reflections on Syria, in the backdrop of Iraq. BMJ Global Health. 2016;1(3):e000149. doi: 10.1136/bmjgh-2016-000149.

15. Iyer AR. Authorship trends in The Lancet Global Health. The Lancet Global health. 2018;6(2):e142. Epub 2018/02/02. doi: 10.1016/s2214-109x(17)30497-7. PubMed PMID: 29389534.

16. Bowsher G, Papamichail A, El Achi N, Ekzayez A, Roberts B, Sullivan R, et al. A narrative review of health research capacity strengthening in low and middle-income countries: lessons for conflict-affected areas. Globalization and health. 2019;15(1):23. Epub 2019/03/28. doi: 10.1186/s12992-019-0465-y. PubMed PMID: 30914049; PubMed Central PMCID: PMCPMC6434620.

17. Ismail SA, McDonald A, Dubois E, Aljohani FG, Coutts AP, Majeed A, et al. Assessing the state of health research in the Eastern Mediterranean Region. Journal of the Royal Society of Medicine. 2013;106(6):224-33. Epub 2013/06/14. doi: 10.1258/jrsm.2012.120240. PubMed PMID: 23761582; PubMed Central PMCID: PMCPMC3705424.

18. Mandil A, El-Jardali F, El-Feky S, Nour M, Al-Abbar M, Bou-Karroum L. Health research institutional mapping: an Eastern Mediterranean Regional perspective. Eastern Mediterranean health journal = La revue de sante de la Mediterranee orientale = al-Majallah al-sihhiyah li-sharq al-mutawassit. 2018;24(2):189-97. Epub 2018/05/12. PubMed PMID: 29748948.

19. Tugwell P, Sitthi-Amorn C, Hatcher-Roberts J, Neufeld V, Makara P, Munoz F, et al. Health Research Profile to assess the capacity of low and middle income countries for equity-oriented research. BMC public health. 2006;6:151. Epub 2006/06/14. doi: 10.1186/1471-2458-6-151. PubMed PMID: 16768792; PubMed Central PMCID: PMCPMC1539005.

20. Gonzalez Block MA, Mills A. Assessing capacity for health policy and systems research in low and middle income countries*. Health Res Policy Syst. 2003;1(1):1. Epub 2003/03/21. PubMed PMID: 12646072; PubMed Central PMCID: PMCPMC151554.

21. Ford N, Mills EJ, Zachariah R, Upshur R. Ethics of conducting research in conflict settings. Conflict and health. 2009;3(1):7. doi: 10.1186/1752-1505-3-7.

Supporting information

S1 Fig. Preferred reporting items for systematic reviews and meta-analyses (PRISMA) study flow diagram for selection.

S2 Fig. Publication year of articles of the papers included in the systematic maps in conflict (N=325) and post-conflict settings

S3 Fig. Types of publication* of the included papers on health care workers in in conflict (N=325) and post-conflict (N=170) settings

S1 File. Search strategy.

S2 File. Definition of themes

Click here to access/download;Figure;S1_Fig. 1.docx

Fig 1. Preferred reporting items for systematic reviews and meta-analyses (PRISMA) study flow diagram for selection.

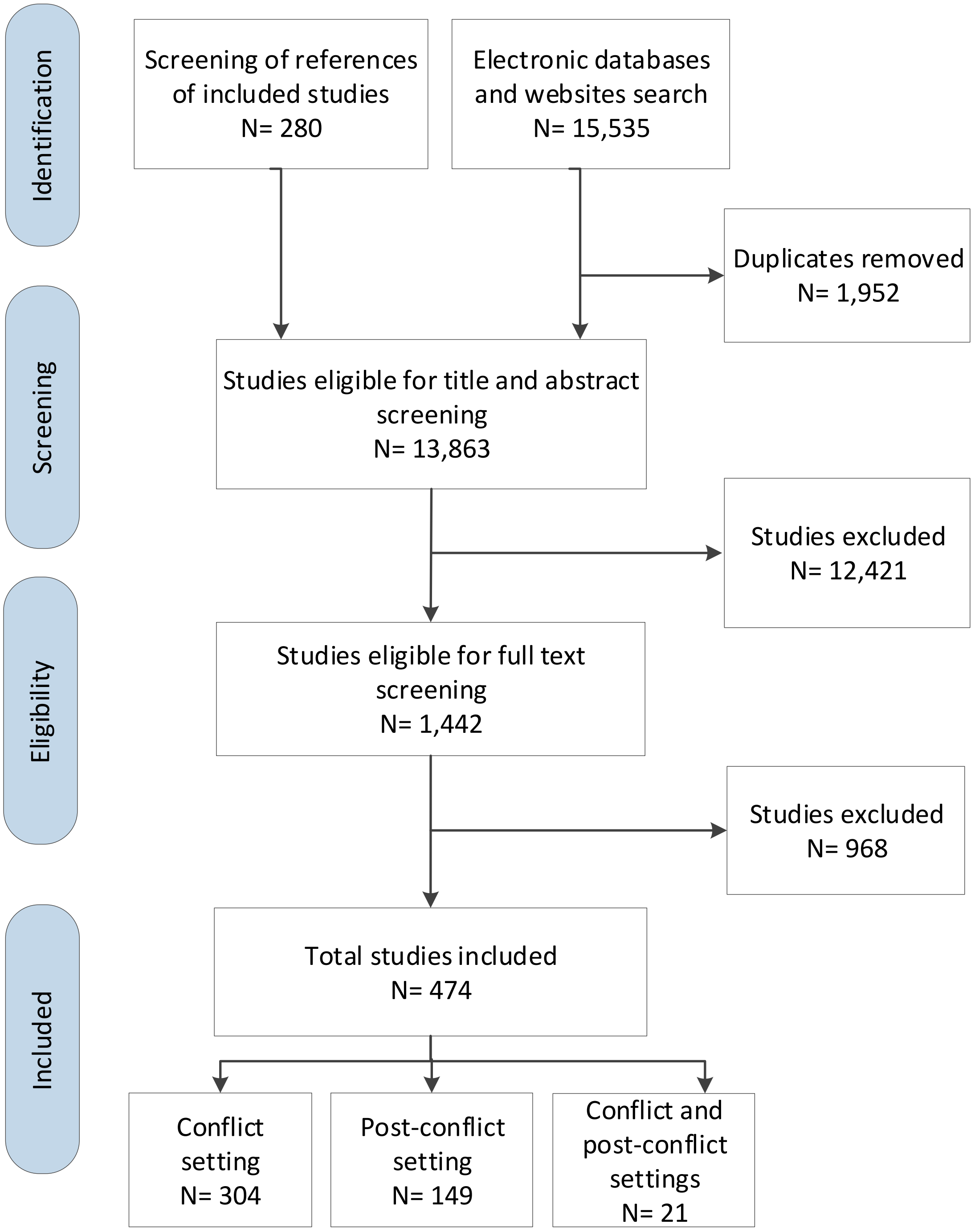

Click here to access/download;Figure;S2_Fig. 2.docx

Fig 2. Publication year of articles of the papers included in the systematic maps in conflict (N=325) and post-conflict settings*

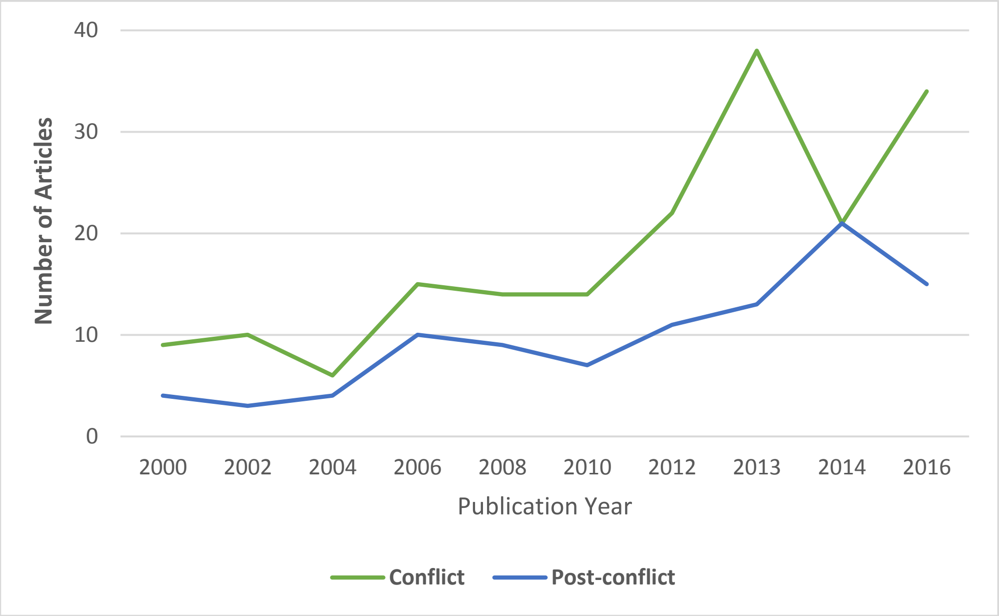

*Data from 2017 was not added to this figure given that the search was performed in June 2017 include editorials and correspondences

Click here to access/download;Figure;S3_Fig. 3.docx

Fig 3. Types of publication* of the included papers on health care workers in in conflict (N=325) and post-conflict (N=170) settings

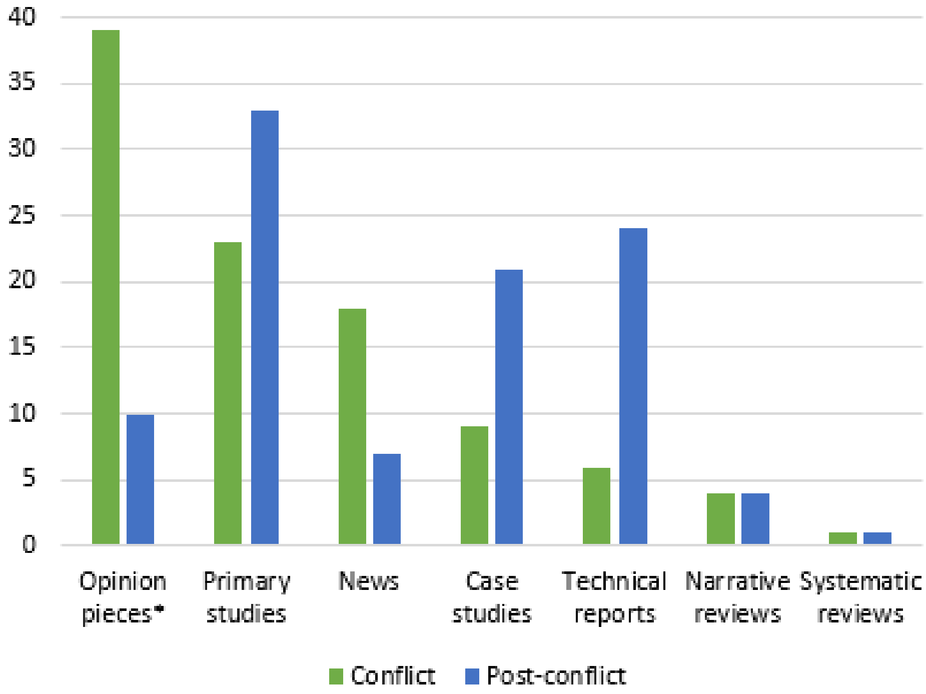

*Opinion pieces include editorials and correspondences

Click here to access/download
Supporting Information
S1_File.docx

Click here to access/download
Supporting Information
S2_File.docx
