# Supplementary material for: Health care workers in conflict and post-conflict settings: Systematic mapping of the evidence
Source: PLoS One. 2020 May 29;15(5):e0233757. doi: 10.1371/journal.pone.0233757 (PMC7259645; doi:10.1371/journal.pone.0233757)
Supplement: Attachment — Submitted filename: HCWs_PLOSONE response letter_20200427 (1).docx [file pone.0233757.s007.docx]

**Department of Internal Medicine**

**American University of Beirut**

27April 2020

Professor Jai K Das

Academic Editor, PLOS ONE

Re: “Health Care Workers in Conflict and Post-Conflict Settings: Systematic Mapping of the Evidence”

Dear Dr. Das,

Thank you for the opportunity to revise and resubmit our above-titled manuscript to PLOS ONE. We sincerely thank you and the Reviewers for the constructive review of our manuscript. Kindly find below a point-by-point response to the comments and a description of the changes made to the manuscript.

We look forward to the outcome of the peer review process.

Sincerely,

Elie A. Akl, MD, MPH, PhD

AUBMC, Department of Internal Medicine

P.O. Box: 11-0236

Riad-El-Solh Beirut 1107 2020

Beirut – Lebanon

Phone: 00961 1 374374

[ea32@aub.edu.lb](mailto:ea32@aub.edu.lb)

The editor’s comments are listed below in **bold**. Our responses to the comments are in regular font. Relevant extracts from the text are in *italic* font and changes are *underlined*.

**We would like to thank the authors for doing a good job and responding to the peer-review comments adequately. However, for the paper to be considered for publication, there are few following areas for the authors to work on.**

- **The major findings (HCW characteristics and themes) are not mentioned in the abstract. Please specify these findings in the abstract.**

**Response:** We thank the Editor for bringing this to our attention. We have added the following to the abstract to highlight major findings about HVWs characteristics and themes:

*Violence against health workers was the most tackled themes of papers reporting on conflict settings while workforce performance was mostly addressed by papers on post-conflict settings. The majority of papers in both conflict and post conflict settings did not report funding sources (81% and 53%), conflicts of interest of authors (73% and 62%) and around half of primary studies did not report on ethical approvals (45% and 41%).*

- **The primary studies included should be referenced in the results section.**

**Response:** Thank you. All necessary changes have been made in the text to include referencing of the included primary studies in the results section (p. 10 of the results section). The included studies can also be found on the interactive gap maps provided in this manuscript.

- **The authors should add a table describing the characteristics of the included studies as an annex (study ID, types of studies, conflict, HCW involvement, any outcomes, any other relevant information)**

**Response:** Thank you for this helpful suggestion. We have added a table as an annex (S4 Appendix) to describe the characteristics of the included studies. We refer to it in the text as follows:

*Fig 2 presents a geographical map of the countries focus of the included papers* *(S2 Fig; S3 Appendix). A description of the included studies is also presented in S4 Appendix.*
